# Preparation 2-hydroxy-1-naphthaldehyde cross-linked Fe_3_O_4_@chitosan-polyacrylamide nanocomposite for removal of everzol black from aqueous solutions

**DOI:** 10.1038/s41598-023-37243-5

**Published:** 2023-06-30

**Authors:** Afshin Saadat, Alireza Banaei, Mehdi Sattarifar, Parinaz Pargolghasemi

**Affiliations:** 1 Department of Chemistry, Germi Branch, Islamic Azad University, Germi, Iran; 2 Department of Chemistry, Payame Noor University, P.O. Box 19395-3697, Tehran, Iran

**Keywords:** Chemistry, Inorganic chemistry, Materials chemistry

## Abstract

In this study, new 2-hydroxy-1-naphthaldehyde linked Fe_3_O_4_/chitosan-polyacrylamide nanocomposite (Fe_3_O_4_@CS@Am@Nph) were prepared. The synthesized nanocomposite was characterized by (FT-IR), X-ray diffraction (XRD), Scanning Electron Microscopy (SEM), vibrating Sample Magnetometry (VSM) and Termogravimetric Analysis (TGA). The 2-hydroxy-1-naphthaldehyde modified Fe_3_O_4_@CS@Am@Nph nanocomposite was used as an effective adsorbent for removal of everzol black from aqueous solutions by batch adsorption procedure. The effects of important parameters on the surface absorption process of everzol black dye, including pH, contact time, adsorbent dosage and initial dye concentration were studied. The Langmuir, Freundlich and Temkin adsorption models were used to describe adsorption isotherms and constants. The equilibrium results revealed that the adsorption behavior of the everzol black dye on the Fe_3_O_4_@CS@Am@Nph nanocomposite fitted well with the Langmuir model. On the basis of the Langmuir analysis, the maximum adsorption capacity (qm) of the Fe_3_O_4_@CS@Am@Nph for everzol black was found to be 63.69 mg/g. The kinetic studies indicated that adsorption in all cases to be a pseudo second-order process. Further, the thermodynamic studies showed the adsorption to be a spontaneous and endothermic process.

## Introduction

The extreme use of colorant substances by industries for decades led to the decline of water bodies in the world^1,2^. For example, industries related dyeing, plasticization and paper making use a lot of water and chemicals to color the products and consequently, they produce a large amount of colored wastewater, which if they are not treated before entering the environment and waters, they will cause many problems. These problems include disrupting the photosynthesis of waters and ecosystems^3^. Moreover, their complex molecular structure and aromatic rings are toxic and carcinogenic, which can affect human health, water microorganisms, and the environment^4,5^. Nanofiltration membranes^6^, ion exchange^7^, electrochemical oxidation^8^, photo-catalytic degradation^9^ and adsorption are methods that have been used to remove colors and pollutants from wastewater. However, most of the above mentioned methods are ineffective due to the factors such as operational costs, secondary wastes, environmental effects and related problems, efficiency and applications^10^. Among these methods, adsorption is more superior than other methods due to the low initial cost, easy design, suitable flexibility and high efficiency^11^. In this field, many absorbents including activated carbon, nanoclays, plant biomass and natural absorbents have been used and reviewed^12^. Among these absorbents, activated carbon is the most suitable absorbent to remove all kinds of pollutants. However, the high price, lack of recycling and reusing have limited the application of this absorbent^13^.

Recently, magnetic nanoparticles have attracted much attentions because they have great magnetic properties such as large surface area, low toxicity, chemical stability, good biocompatibility and biodegradation^14,15^. It can also be separated from aqueous solutions easily and quickly by using an external magnetic field without requiring tedious filtration or centrifugation^16^. Chemical or physical change of the surface Fe_3_O_4_ nanoparticles with some surfactants or polymers is required for improving the adsorption performance of Fe_3_O_4_ nanoparticles^17^.polysaccharides such as chitosan and its derivatives are more interesting, since the use of chitosan based adsorbents is one of the best ways to remove the colors and ions of heavy metals even at low concentrations^18^. Chitosan mainly contains poly2-deoxy-d-glucose which is a biopolymer derivative and has well known polymer properties. It has attracted scientist’s attentions because of biocompatibility, biodegradability and nontoxic properties^19–22^. Because chitosan contains high amounts of amine and hydroxyl groups, it has a very high absorption ability to remove many types of metals such as copper, chromium, silver and platinum. However, in order to improve the absorption properties of adsorbents, much attentions have been paid to the design and synthesis of new adsorbents. For example, magnetic chitosan complex coated on the surface Fe_2_O_3_ has been used for removing alizarin red from water environments^23^. Wang et al. employed magnetic polydopamine-chitosan nanoparticles as adsorption material for the removal of Methylene blue and Malachite green from aqueous solutions^24^. Zhu et al. synthesized the chitosan-modified magnetic graphitized multi-walled carbon nanotubes for the effective removal of Congo red from aqueous solution^25^. Armagan et al. performed a comprehensive study on the removal of everzol black by Zeolite^26^.

**Figure 1:**
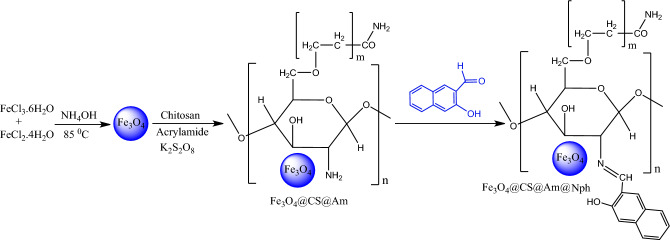
The synthesis route of Fe_3_O_4_@CS@Am@Nph nanocomposite.

In this study, new 2-hydroxy-1-naphthaldehyde linked Fe_3_O_4_/chitosan-polyacrylamide nanocomposite was synthesized (Fig. 1). The nanocomposite prepared was applied for the removal of the Everzol black from aqueous solution. Moreover, the effects of various parameters such as pH, adsorbent dosage, initial dye concentration and contact time on adsorption behavior were studied. Adsorption isotherms, kinetics and thermodynamic studies have been reported to account for the nature of adsorption process.

## Results and discussion

### Preparation of Fe_3_O_4_@CS@Am@Nph nanocomposite

In the present study, for the preparation of Fe_3_O_4_@CS@Am@Nph nanocomposite two-step method was successfully used. In the first step, the Fe_3_O_4_@CS@Am nanoparticles were prepared by reaction of Fe_3_O_4_ nanoparticles, chitosan and Potassium persulfate. In the second step, Fe_3_O_4_@CS@Am nanoparticles were connected on the surface of 2-hydroxy-1-naphthaldehyde by the formation of a Schiff base bond between the amine groups of chitosan and the carbonyl group of 2-hydroxy-1-naphthaldehyde. The synthesis route of Fe_3_O_4_@CS@Am@Nph adsorbent are shown in Fig. 1.

### FT-IR analysis

**Figure 2:**
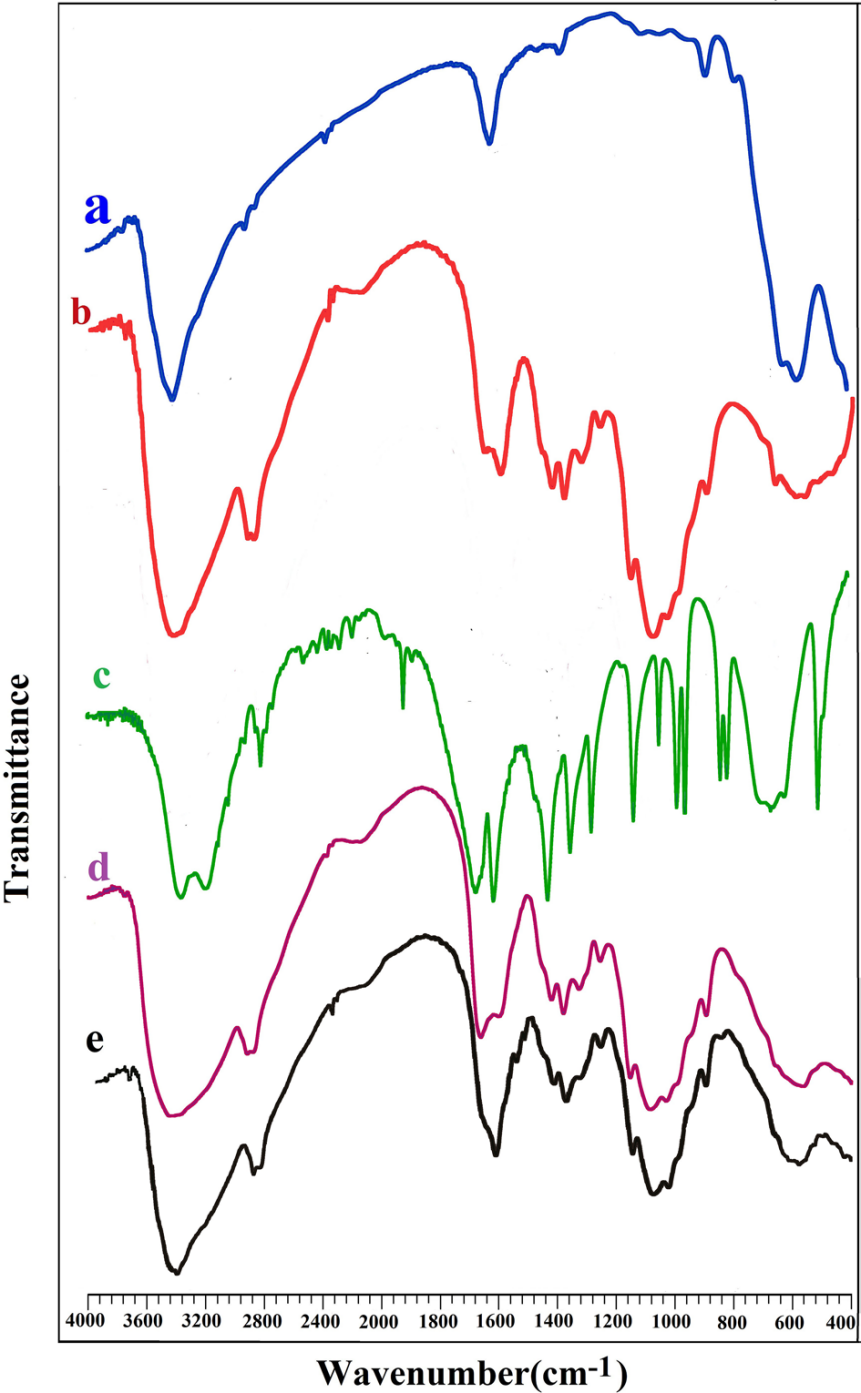
FT-IR spectra of (a) Fe_3_O_4_, (b) Chitosan, (c) Acrylamide, (d) Fe_3_O_4_@CS@Am, (e) Fe_3_O_4_@CS@Am@Nph.

FT-IR spectra of Fe_2_O_3_, chitosan, acrylamide, Fe_3_O_4_@CS@Am and Fe_3_O_4_@CS@Am@Nph are shown in Fig. 2. The characteristic peaks (blue line) of the Fe_2_O_3_ appeared at 582 and 628 cm^−1^ corresponding to Fe–O stretching vibration 1628 and 3426 cm^−1^ and the peaks at 1628 and 3426 cm^−1^ assigned to OH bending vibration of Fe_2_O_3_ respectively^27^. For chitosan (red line), a broad band around 3425 cm^−1^ belongs to amino (NH_2_) and hydroxyl (OH) groups. Beside the peaks at 2916 and 1381 cm^−1^ assign to C–H and C–N respectively^28^. The FTIR spectra of acrylamide (green line) demonstrated absorption peak at 1674 cm^−1^ showed the presence of C=O group of amides^29^, also the peaks at 3352, 3192 and 2812 cm^−1^ attributed to N–H and C–H stretching vibration respectively. The spectrum of Fe_3_O_4_@CS@Am (Fig. 2d) showed broader band at 3442 cm^−1^ which belonged to O–H stretching vibration. Furthermore, the peaks appearing at 2916 cm^−1^ and 2879 cm^−1^ belonged to C–H stretching of the alkyl group. This spectrum also showed that the peaks 1662, 1598 and 565 cm^−1^ are attributed C=O (amide), N–H and Fe–O bands, respectively. The FT-IR spectrum of the Fe_3_O_4_@CS@Am@Nph (Fig. 2e) showed a peak at 1627 cm^−1^ resulted from C=N vibration, which can be due to the of the formed Schiff base between the remained free amino groups of chitosan and 2-hydroxy-1-naphthaldehyde.

### XRD analysis

**Figure 3:**
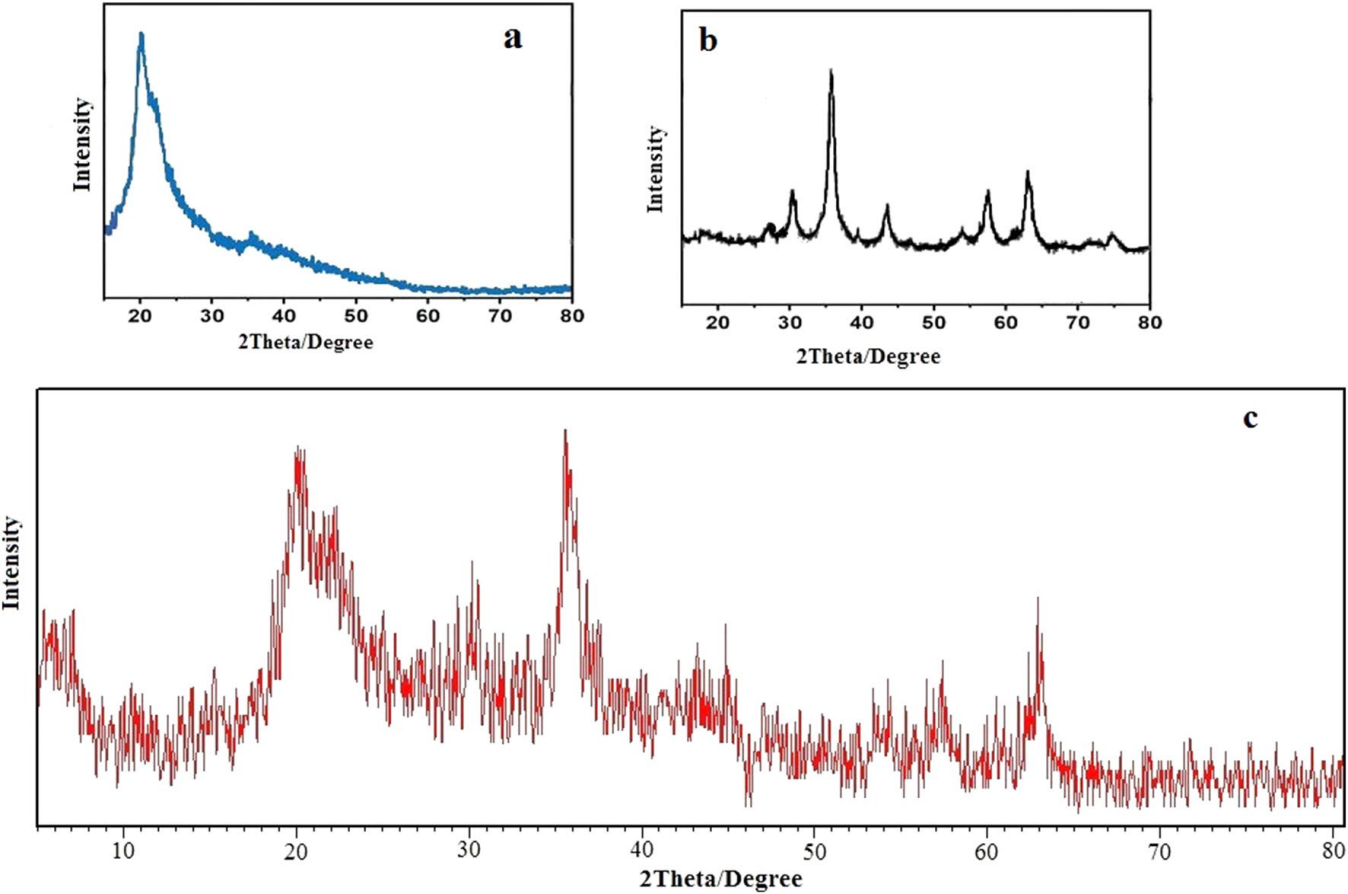
XRD pattern of (**a**) Chitosan, (**b**) Fe_3_O_4_ and (**c**) Fe_3_O_4_@CS@Am@Nph nanocomposite.

X-ray diffraction of chitosan, Fe_3_O_4_ nanoparticles and Fe_3_O_4_@CS@Am@Nph nanocomposite particles are shown in Fig. 3. The characteristic XRD peaks for Fe_3_O_4_@CS@Am@Nph observed at 2θ = 30.3° (220), 35.6° (311), 43.5° (400), 53.5° (422), 57.3° (511) and 62.5° (440) belong Fe_3_O_4_ nanoparticles. Beside the peaks at 2θ = 20° are related to chitosan structure^30–32^. The average size of Fe_3_O_4_@CS@Am@Nph nanocomposite particles is also estimated via Debye–Scherer equation:

$$D=\frac{K \lambda }{\beta \cos\theta },$$

where D is the average size, λ is the X-ray source wavelength (1.54 Å), β is the full width at half maximum (FWHM) of the diffraction peak and θ is the Bragg’s angle.

According to the Debye–Scherrer equation, the particles size of the Fe_3_O_4_@CS@Am@Nph nanocomposite was 193 nm.

### SEM analysis

**Figure 4:**
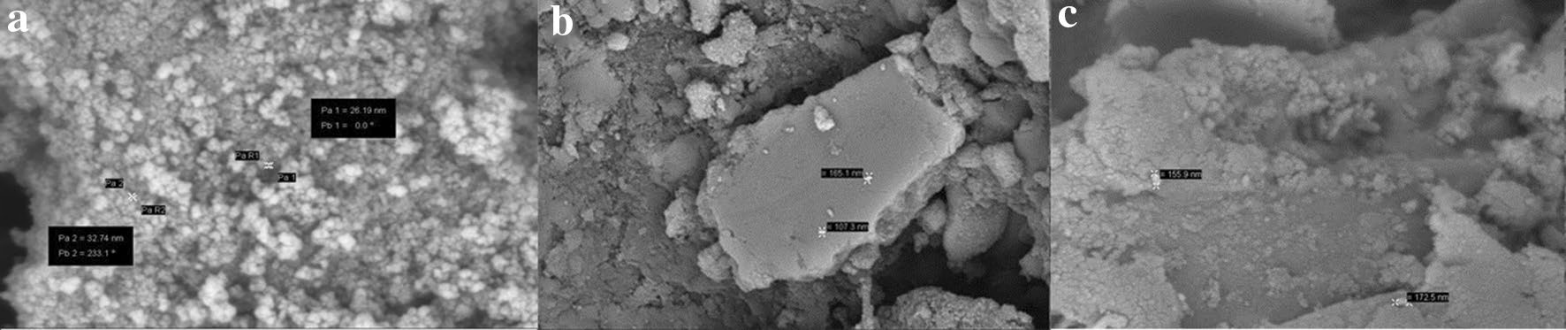
SEM images of (**a**) Fe_3_O_4_, (**b**) Fe_3_O_4_@CS@Am and (**c**) Fe_3_O_4_@CS@Am@Nph nanocomposite.

Scanning electron microscopy (SEM) is used to characterize the morphology and size of Fe_3_O_4_, Fe_3_O_4_@CS@Am and Fe_3_O_4_@CS@Am@Nph nanocomposite. As shown in Fig. 4 the morphology of nanoparticles obtained nearly spherical shape. Furthermore, the size of Fe_3_O_4_, Fe_3_O_4_@CS@Am and Fe_3_O_4_@CS@Am@Nph nanocomposite are relatively uniform and the average diameter are 26–32, 107–165 and 155–173 nm respectively.

### TGA analysis

The TGA curve of the Fe_3_O_4_@CS@Am is shown in Fig. S.1. The TGA of the Fe_3_O_4_@CS@Am displayed three stages of weight loss between 26 and 600 °C. The first stage decomposition occurred between 26 and 230 °C with 10% corresponds to the adsorbed and bound water in the sample^33^. The second stage of weight loss was observed in the temperature ranges of 230–315 °C associated with weight loss 31% is related to the heat decomposition of chitosan structure. And the loss 43% in the range from 315 to 580 °C in the third stage is attributed to the decomposition of cross-linked chains of polyacrylamide. About 16% of the sample retained at 600 °C attributed to the existence of Fe_3_O_4_ nanoparticles. Furthermore, the TGA of the Fe_3_O_4_@CS@Am@Nph nanocomposite (Fig. S.2) showed three stages of weight loss between 26 and 600 °C. The first stage decomposition occurred between 30 and 23 °C with 11% assigned to the adsorbed water in the sample. In two and third stage between 226 and 600 °C weight loss 77% was observed which is attributed to the decomposition of the anchored organic polymers of the adsorbent. The content of Fe_3_O_4_ nanoparticles in the nanocomposite is about 12%.

### Brunauere–Emmette–Teller (BET)

The BET analysis was used to determine the surface area, pore size, and pore volume of the Fe_3_O_4_@CS@Am@Nph nanocomposite. Figure S.2 represents the BET nitrogen adsorption/desorption isotherm curve of the Fe_3_O_4_@CS@Am@Nph nanocomposite. The surface area, pore volume and pore diameter were found to be 9.47 (m^2^/g), 0.031 (cm^3^/g) and 13.23 nm respectively for Fe_3_O_4_@CS@Am@Nph nanocomposite. The isotherm curve closely matches to a typical type V isotherm graph confirming the mesoporous property of the nanocomposite^34^.

### Magnetization analysis

**Figure 5:**
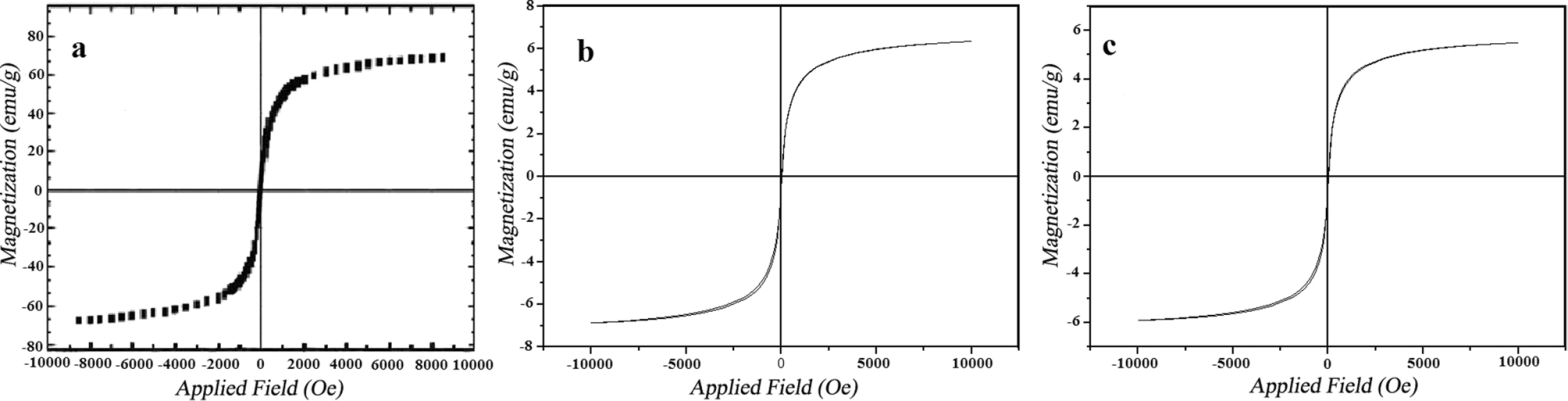
Hysteresis loops of (**a**) Fe_3_O_4_, (**b**) Fe_3_O_4_@CS@Am and (**c**) Fe_3_O_4_@CS@Am@Nph nanocomposite at room temperature using VSM.

The magnetic moment of the prepared Fe_3_O_4_@CS@Am@Nph nanocomposite was measured over a range of applied fields between 10,000 and − 10,000 Oe. The magnetization curves of the Fe_3_O_4_, Fe_3_O_4_@CS@Am and Fe_3_O_4_@CS@Am@Nph at room temperature are shown in Fig. 5. The VSM results indicate coating the surface of the magnetite nanoparticles with acrylamide, chitosan and 2-hydroxy-1-naphthaldehyde leads to a decrease in the saturation magnetization. This is due to the presence of acrylamide, chitosan and 2-hydroxy-1-naphthaldehyde on the surface of Fe_3_O_4_ nanoparticles which may generate a magnetically dead layer so any crystalline disorder within the surface layer cause to a significant decrease in the saturation magnetization of nanoparticles^35^. The saturation magnetization values for the Fe_3_O_4_ particles, Fe_3_O_4_@CS@Am and Fe_3_O_4_@CS@Am@Nph nanocomposite were 67, 7and 6 emu/g, respectively.

### Sorption studies of selected dyes

#### Effect of adsorbent dosage

**Figure 6:**
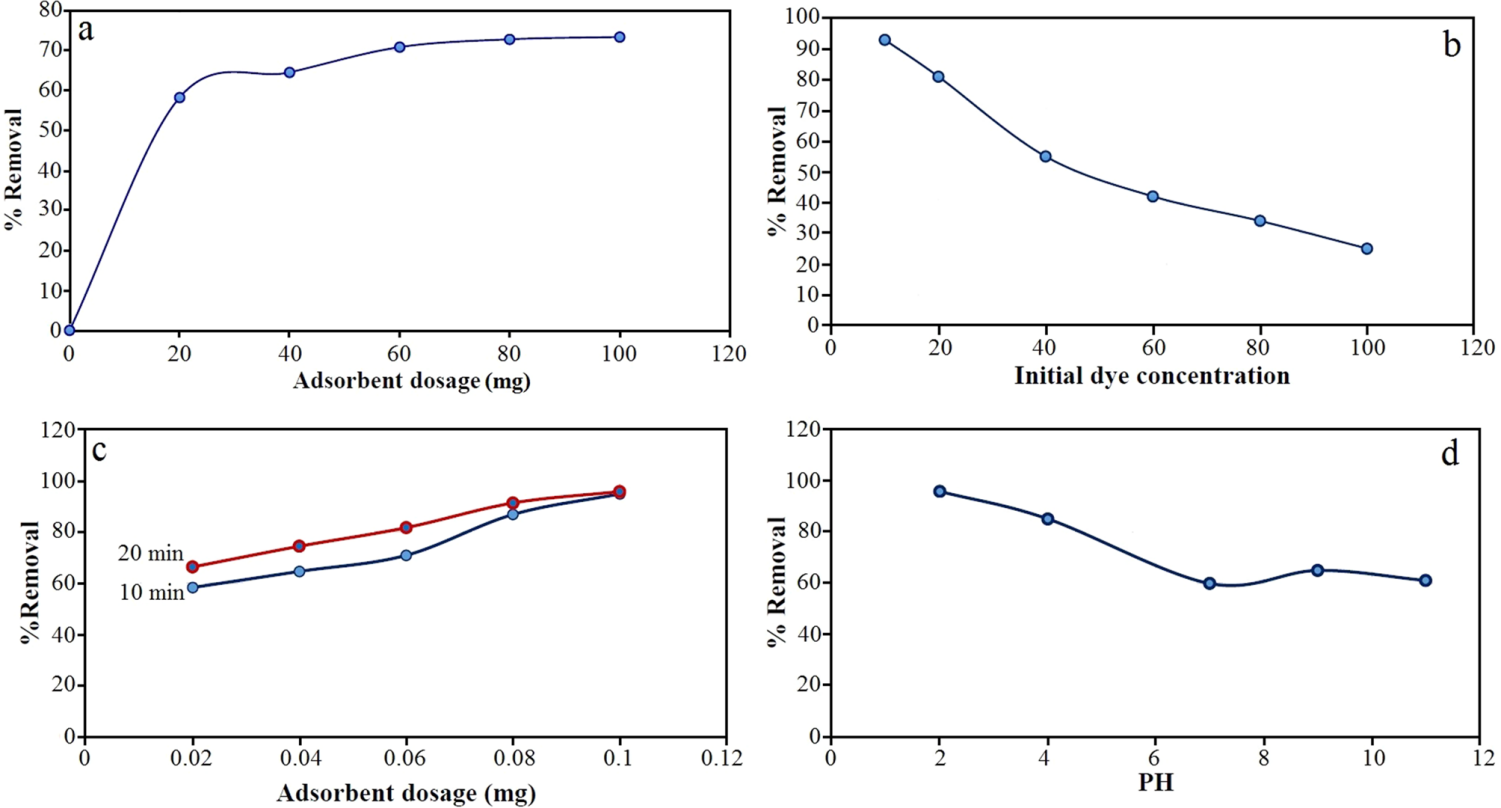
Effect of adsorbent dose (**a**), initial dye concentration (**b**), contact time (**c**) and pH solution (**d**) on removal of everzol black dye by Fe_3_O_4_@CS@Am@Nph (200 rpm, 25 °C).

One of the important factors which affects adsorption processes is adsorbent dose since it determines the capacity of adsorbent for a given initial concentration of dye solution^36^. In this study, the influence of adsorbent dose on adsorption removal of everzol black dye was studied by using different amounts of sorbent (i.e. 20, 40, 60, 80 and 100 mg) in 40 mL of 100 mg/L solution of dye at 25 °C for 10 min. Figure 6a showed effect of adsorbent dosage on the percentage removal of dye. The results showed that the percent sorption of the everzol black dye increased by increasing the dosage of adsorbent. With the increase in dosage of Fe_3_O_4_@CS@Am@Nph nanocomposite, the percentage removal of everzol black dye increased from 58.25 to 94.87. Percentage removal increase can be related to the increased surface area of the adsorbent and availability of more adsorption sites. Therefore, 60 mg adsorbent dosage was chosen for the further experiments.

#### Effect of initial dye concentration

The Effect of initial concentration of everzol black on adsorption of it on Fe_3_O_4_@CS@Am@Nph nanocomposite were studied in different initial concentrations of dye between 10 and 100 mg/L with keeping constant the other parameters. As result of Fig. 6b illustrates, the percent of dye removal decreases with increase in dye initial concentration from 10 to 100 mg/L. This may be due to the increase of enough number of active sites of dye molecules for binding on the surface of the adsorbent. The percentage removal of everzol black decreases from 93.2 to 25.4%.

#### Effect of contact time

The effect of contact time on adsorption of everzol black the surface of Fe_3_O_4_@CS@Am@Nph nanocomposite were studied at room temperature with the different contacting time at 10 and 20 min. As it can be seen in Fig. 6c, by increasing the contact time percent adsorption of everzol black on Fe_3_O_4_@CS@Am@Nph was increased.

#### Effect of initial pH solution

The pH plays a crucial role in the adsorption of dye onto the adsorbent. Indeed, the pH affects the adsorption process through the degree of ionization, the surface charge of the adsorbent, or the speciation of the adsorbate. In this study, the effect of initial pH on the sorption of everzol black onto Fe_3_O_4_@CS@Am@Nph nanocomposite were studied at different values from 2 to 12. For this experiment, 0.1 M NaOH and 0.1 M HCl solutions were used to adjust the pH of the solution. The effect of pH on the percentage removal of everzol black by Fe_3_O_4_@CS@Am@Nph is shown in Fig. 6d. In acidic conditions the amount of adsorption is increased that can be due to electrostatic attraction between positive charge of amino groups of chitosan and negative charge of sulfonate groups of the everzol black dye.

### Adsorption isotherms

**Figure 7:**
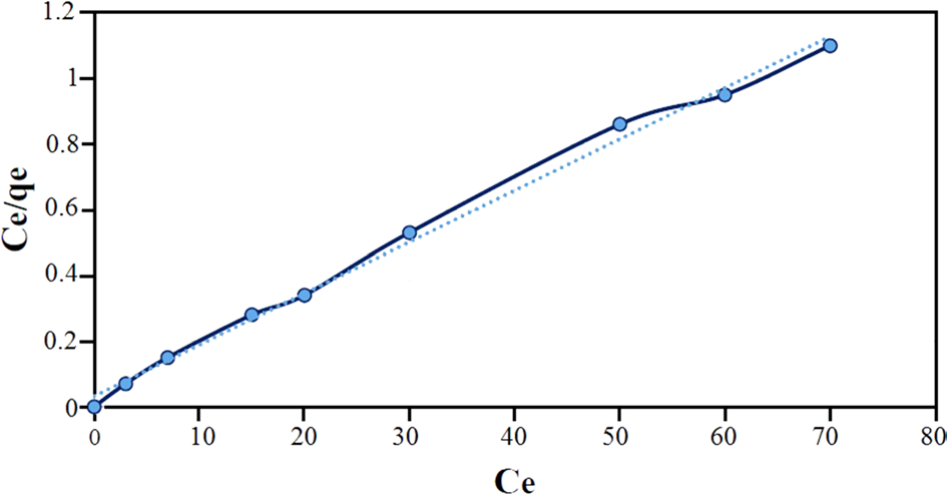
Langmuir plot for the adsorption of everzol black (200 rpm, 25 °C and pH 7).

Adsorption isotherm is a method to investigate the relationship between the adsorbed amount in the liquid phase on adsorbent in equilibrium and constant temperature^37^. In fact, the adsorption isotherm describes the interaction between the adsorbent and adsorbed surfaces. Therefore, it is always considered as a fundamental factor for determining the absorbent capacity and optimizing the absorbents^38^. In the present study, Langmuir, Freundlich and Temkin isotherm models were used to obtain the isotherm parameters for adsorption of everzol black onto Fe_3_O_4_@CS@Am@Nph nanocomposite. Investigating the experimental data obtained from adsorption in equilibrium with theoretical models and obtaining the relationship between them provides important information for the best possible design of an absorbent system. Langmuir adsorption isotherm: In this model, there is no interaction among adsorbed molecules and adsorption process happens on homogeneous surfaces, showed in below Eq. (1)^39^:

1 $$\frac{{\text{C}}_{\text{e}}}{{\text{q}}_{\text{e}}}=\frac{1}{{\text{K}}_{\text{L}}\cdot {\text{q}}_{\text{m}}}+\frac{{\text{C}}_{\text{e}}}{{\text{q}}_{\text{m}}},$$

where, C_e_ is the equilibrium concentration of the dye solution (mg/L), q_e_ (mg/g) is the amount of dye adsorbed, q_m_ is the value of monolayer adsorption capacity in Langmuir model and K_L_: constant value of Langmuir (mg/L). The Langmuir plot for the adsorption of everzol black onto Fe_3_O_4_@CS@Am@Nph nanocomposite at different temperatures is shown in Fig. 7.

Freundlich isotherm model (2) is the more for the adsorption of components dissolved in a liquid solution, it is assumed that: First, the adsorption is monolayer and chemical, and second, the energy of the adsorption sites is not the same, i.e. the adsorbent surface is not uniform^40^:

2 $$\text{Ln }{q}_{e}=\text{Ln }{\text{K}}_{\text{f}}+\left(\frac{1}{\text{n}}\right) \text{Ln }{\text{C}}_{\text{e}}.$$

**Figure 8:**
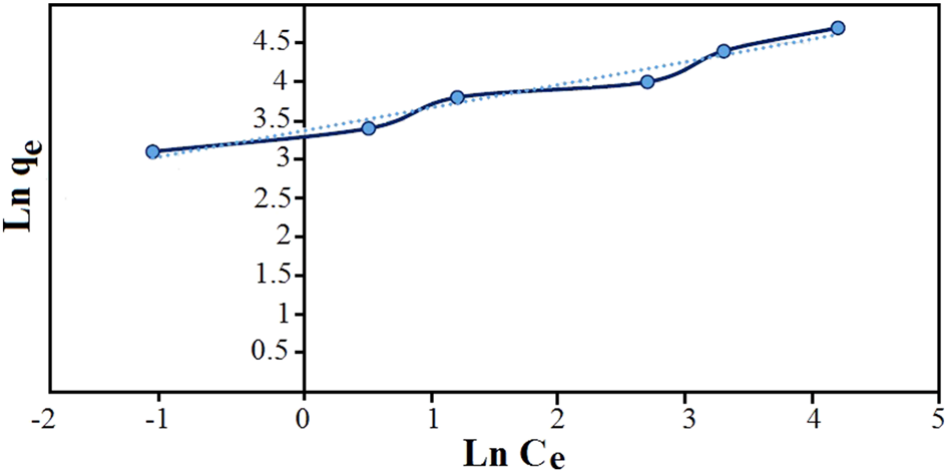
Freundlich plot for the adsorption of everzol black (200 rpm, 25 °C and pH 7).

K_F_ and n are experimental constants where K_F_ is adsorption capacity at unit concentration (L/mg) and n shows the intensity of adsorption. The 1/n values can be classified as irreversible (1/n = 0), favorable (0 < 1/n < 1) and unfavorable (1/n > 1). Calculation of K_F_ and n in Freundlich model for Fe_3_O_4_@CS@Am@Nph nanocomposite shown in Fig. 8. Also, the separation factor (R_L_) was calculated by the following Eq. (3):

3 $${\text{R}}_{\text{L}}=\frac{1}{1+{\text{K}}_{\text{L}}\cdot {\text{C}}_{0}}.$$

The values of R_L_ can illustrate the shape of the isotherm to be either unfavorable (R_L_ > 1), linear (R_L_ = 1), favorable (0 < R_L_ < 1) or irreversible (R_L_ = 0). The values of Langmuir and Freundlich parameters and the regression coefficients R^2^ of the adsorption of everzol black onto Fe_3_O_4_@CS@Am@Nph are given in Table S.1. According to Table S.1, the value of R_L_ was obtained in the range of 0 < R_L_ < 1, that showed adsorption of the everzol black on Fe_3_O_4_@CS@Am@Nph was favorable. The maximum monolayer adsorption capacity (qm) calculated by Langmuir model was found to be 63.69 and regression coefficient value is 0.9959.

**Figure 9:**
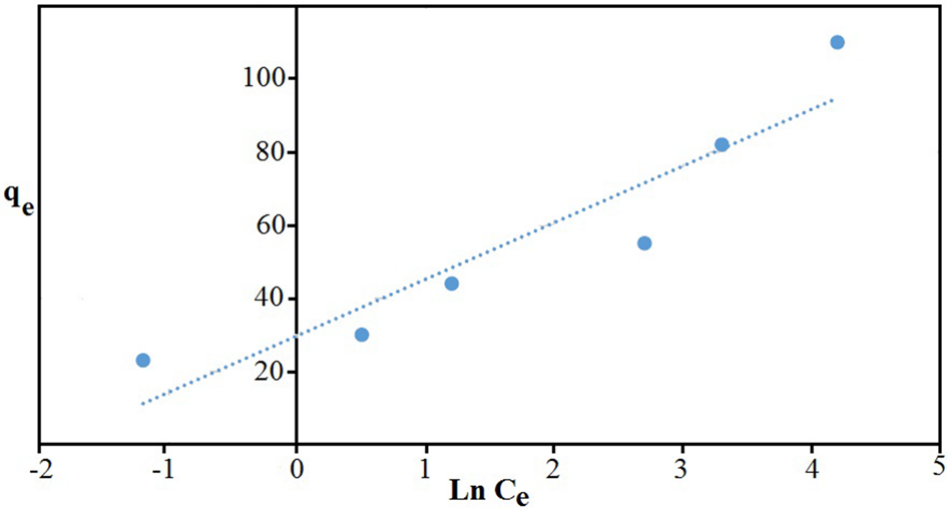
Temkin plot for the adsorption of everzol black (200 rpm, 25 °C and pH 7).

Temkin adsorption isotherm directly takes into account of adsorbent-adsorbate interactions. The Temkin isotherm equation is:

4 $${\text{q}}_{\text{e}}=\text{BlnA}+{\text{BlnC}}_{\text{e}},$$

5 $${{\text{B}}}=\frac{{\text{RT}}}{{\text{b}}},$$

where R is gas constant 8.314 J/mol/K. T is absolute temperature (K), b is the Temkin constant related to the heat of adsorption (J/mol) and A is the equilibrium binding constant corresponding to the maximum binding energy (L/g). The linear plot (Fig. 9) of q_e_ versus lnC_e_ enable to determine the constant A and b. The values of Temkin parameters and the regression coefficients R^2^ of the adsorption of everzol black onto Fe_3_O_4_@CS@Am@Nph are given in Table S.1.

### Adsorption kinetics

**Figure 10:**
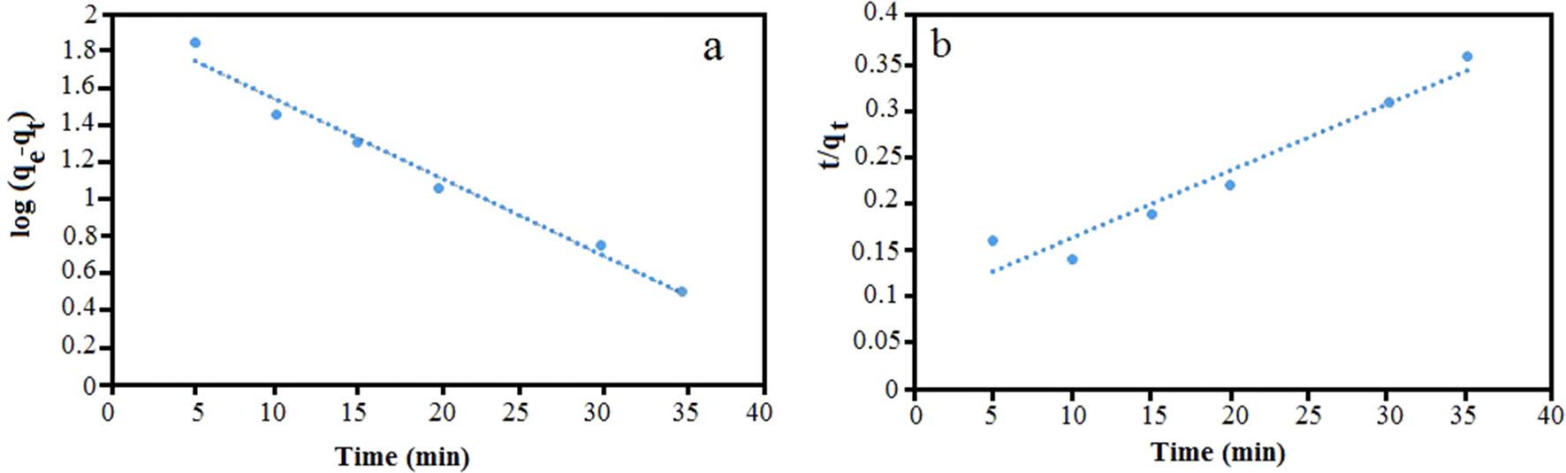
Pseudo-first-order (**a**) and Pseudo-Second-order (**b**) model for the removal kinetics of everzol black on Fe_3_O_4_@CS@Am@Nph nanocomposite (50 mg/L, 25 °C and pH 7).

In order to determine the type of adsorption kinetics pseudo-first-order^41^ and pseudo-second-order^42^ kinetics were investigated for the Fe_3_O_4_@CS@Am@Nph nanocomposite. The linear equation of pseudo-first-order and pseudo-second-order kinetic are given by Eqs. (6) and (7), respectively:

6 $$\text{log}\left({\text{q}}_{\text{e}}-{\text{q}}_{\text{t}}\right)={\text{logq}}_{\text{e}}-\frac{{\text{K}}_{1}}{2.303}\text{t},$$

7 $$\frac{\text{t}}{{\text{q}}_{\text{t}}}=\frac{1}{{\text{K}}_{2}{\text{q}}_{\text{e}}^{2}}+\frac{\text{t}}{{\text{q}}_{\text{e}}},$$

where q_e_ and q_t_ (mg/g) is the amount of dye adsorbed at equilibrium and at time t, K_1_ and K_2_ (min^−1^) are the rate constants. Figure 10 shows the absorption kinetics using different models. In the pseudo-first-order model, the values of rate constant k_1_ and q_e_ are calculated from the straight line plots of log(q_e_–q_t_) vs time (Fig. 10a). The values of first order rate constant (k_1_), amount of dye adsorbed at equilibrium (q_e_) and coefficient of linear regression (R^2^) were obtained 0.012 min^−1^, 28.8 (mg/g) and 0.9566, respectively. As it is shown in the Fig. 10b pseudo-second-order constants can be calculated from the linear plot between t/qt and time. The values of k_2_, q_e_ and R^2^ were obtained 0.0094/min, 33.22 (mg/g) and 0.9918, respectively. The q_e_ value obtained by calculating pseudo second order kinetic is close to the experimental value (49.73), also the pseudo second order model has high regression coefficient (R^2^ = 0.9918) than the pseudo first order (R^2^ = 0.9566).

### Thermodynamic studies

**Figure 11:**
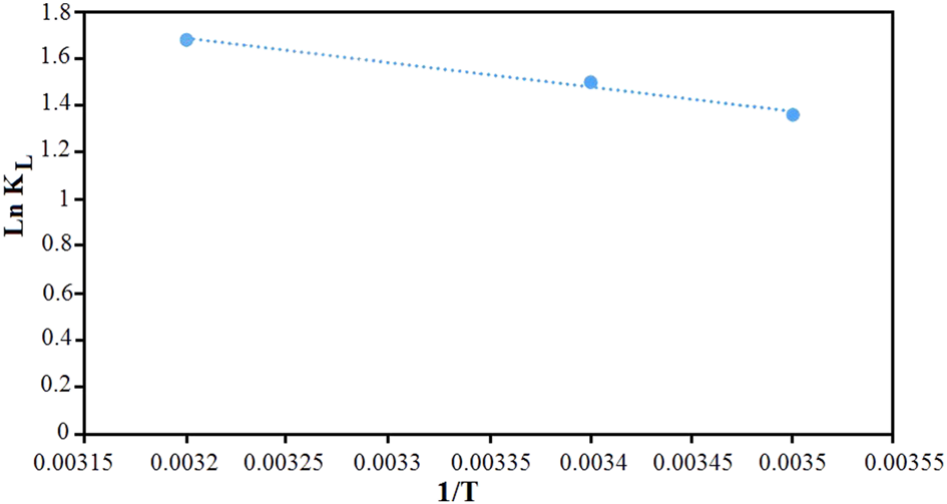
Thermodynamic plot for removal of everzol black on Fe_3_O_4_@CS@Am@Nph nanocomposite.

In order to investigate the thermodynamics of adsorption, important parameters such as entropy change (ΔS), enthalpy change (ΔH) and standard Gibbs free energy change (ΔG) on the adsorbent at different temperatures (283, 293 and 308 K) were investigated for surface adsorption of everzol black dye. The values of thermodynamic relations of adsorption were calculated using the following equations:

8 $$\text{Ln }{\text{K}}_{\text{L}}=\frac{{\Delta \text{S}}^{\circ }}{\text{R}}-\frac{{\Delta \text{H}}^{\circ }}{\text{RT}},$$

9 $${\Delta \text{G}}^{^\circ }={\Delta \text{H}}^{^\circ }-\text{T}{\Delta \text{S}}^{^\circ },$$

where K_L_ is the Langmuir constant (L/mol), T is the solution temperature and R is the universal gas constant (8.314 J/mol K). The values of enthalpy changes of adsorption $({\Delta \text{H}}^{^\circ })$ and entropy changes ${(\Delta \text{S}}^{^\circ })$ were determined from slope and intercept of plot Ln K_L_ vs 1/T (Fig. 11). Table S.2 shows the thermodynamic parameters for the adsorption of everzol black on Fe_3_O_4_@CS@Am@Nph nanocomposite. The positive value of ${\Delta \text{H}}^{^\circ }$ shows that the adsorption of everzol black on Fe_3_O_4_@CS@Am@Nph nanocomposite is endothermic. The increasing the degree of freedom of the everzol black on the nanocomposite may be its reason. Also, The positive value of ${\Delta \text{S}}^{^\circ }$ indicate the increased randomness and disorder at the adsorbent-solution interface during the adsorption of dye on Fe_3_O_4_@CS@Am@Nph nanocomposite. The negative values of ${\Delta \text{G}}^{^\circ }$ in different temperatures shows that the adsorption of dye on Fe_3_O_4_@CS@Am@Nph nanocomposite is spontaneous process.

### Adsorption mechanism

**Figure 12:**
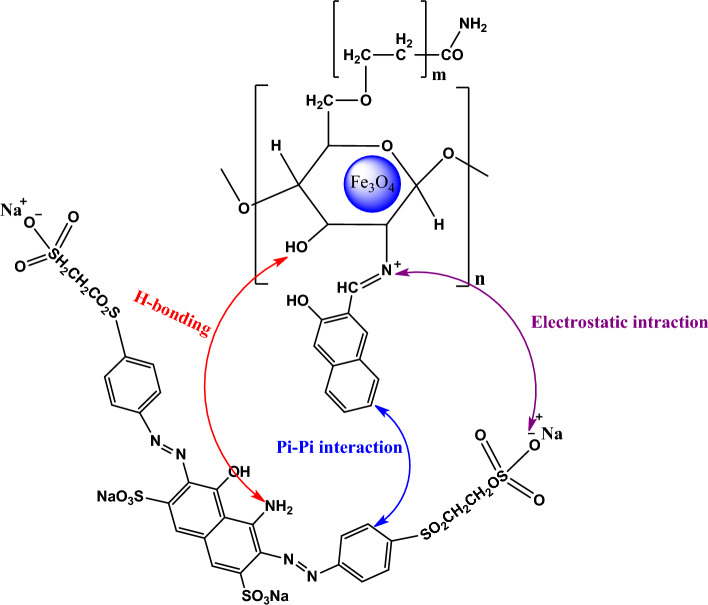
Mechanism of everzol black adsorption on Fe_3_O_4_@CS@Am@Nph nanocomposite.

Figure 12 shows mechanism of adsorption of everzol black on Fe_3_O_4_@CS@Am@Nph nanocomposite. As seen in Fig. 11, the π–π bond interactions between aromatic rings of dye and 2-hydroxy-1-naphthaldehyde, the electrostatic interactions of negatively charged sulfonate groups of dye and the positively charged protonated amino groups of chitosan and also hydrogen bonding interactions between amine groups and oxygen atom of OH group play important role in adsorption of everzol black on Fe_3_O_4_@CS@Am@Nph nanocomposite.

### Reusability studies

**Figure 13:**
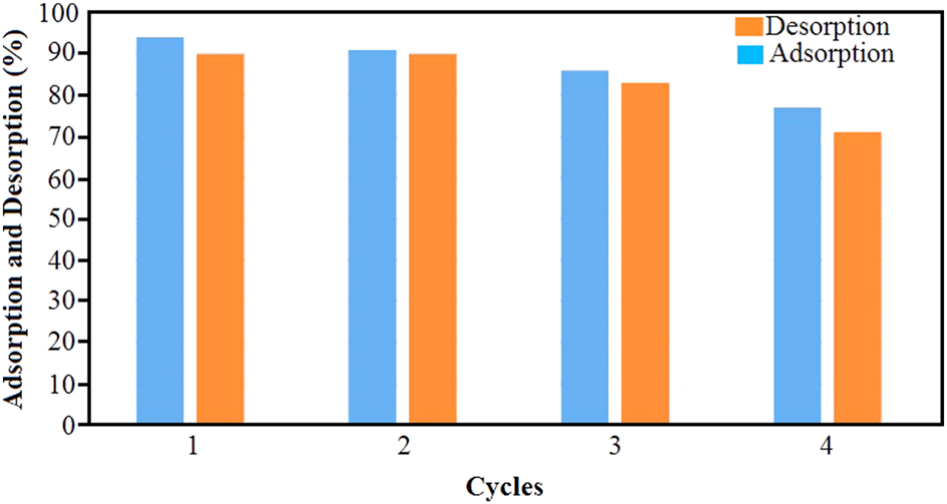
Regeneration studies for the adsorption–desorption of dye onto Fe_3_O_4_@CS@Am@Nph nanocomposite.

The reusing of adsorbent is of great importance as a cost effective process in water treatment. The regeneration ability of Fe_3_O_4_@CS@Am@Nph sample was evaluated by studying adsorption–desorption process in four cycle. Figure 13 shows the percentage removal of dye in 0.1 M HCl solution. As can be seen from Fig. 13, after 4 successive cycles, the dye removal percentage decreased slightly and was still 71%. This suggested that the Fe_3_O_4_@CS@Am@Nph nanocomposite is efficient for everzol black.

### Comparison with other reported adsorbents

The result obtained by comparing this adsorbent with other established adsorbents were shown in Table 1. As Table 1 demonstrates, that the Fe_3_O_4_@CS@Am@Nph nanocomposite had an acceptable adsorption capacity for everzol black dye in comparison with other adsorbents. The high adsorption capacity of studied adsorbent reveals that the adsorbent is very effective in the removal of everzol black from aqueous solutions.

**Table 1:** Comparison of the adsorption capacity of present system with other reported systems.

Adsorbents	Dye	q_m_ (mg/g)	References
ChM nanoparticles	Reactive Black 5	50.00	^43^
ChM GL nanoparticles	Reactive Black 5	36.94	^43^
ChM ECH nanoparticles	Reactive Black 5	33.63	^43^
CS@ZnO-MS nanocomposite	Eriochrome Black-T	30.00	^44^
CS@ZnO-MA nanocomposite	Eriochrome Black-T	26.45	^44^
Fe_3_O_4_@CS@Am@Nph nanocomposite	Everzol black	63.69	Present study

## Conclusion

In this study, 2-hydroxy-1-naphthaldehyde linked Fe_3_O_4_/chitosan-polyacrylamide nanocomposite was prepared. The synthesized nanoparticles were characterized by (FT-IR)*,* XRD, SEM, VSM and TGA. The modified Fe_3_O_4_/chitosan-polyacrylamide nanocomposite was used successfully as an effective sorbent for the removal of everzol black dye from aqueous solutions. The effects of various parameters such as adsorbent dose, solution pH, initial dye concentration and contact time on the adsorption process were investigated. The Langmuir, Freundlich and Temkin isotherm models were applied to analyze the experimental data. The maximum adsorption capacity of Fe_3_O_4_@CS@Am@Nph for everzol black was 63.69 mg/g at 25 °C. The kinetic studies indicated the adsorption in all cases to be a pseudo second-order process. Further, the thermodynamic studies showed the adsorption to be a spontaneous and endothermic process.

## Experimental

### Chemicals and reagents

Ferric chloride hexahydrate (FeCl_3_⋅6H_2_O) with 98% purity, ferrous chloride tetrahydrate (FeCl_3_⋅4H_2_O) with 98% purity, absolute ethanol, 2-hydroxy-1-naphthaldehyde, Chitosan, glycerol with 99% purity and ammonia (NH_3_) with 25% purity were purchased from Merck, Germany. Everzol Black (chemical formula = C_26_H_21_N_5_Na_4_O_19_S_6_, Molecular weight (g/mol) = 991.82, λ_max_ = 436 nm) was purchased from the Textile Factory. The chemical structure of Everzol Black is shown in Fig. S.3.

### Instrumentation

FT-IR spectra (Shimadzu prestige-21) were used to determine the identity of the as prepared nanoparticles and to characterize the coated Fe_3_O_4_ nanoparticles. X-ray powder diffraction measurements were performed using an X-ray diffractometer (XRD) (Perkin Elmer) at ambient temperature. The surface morphology of the silica-supported ligands was identified with a scanning electron microscope (LECO SEM, Michigan, USA). Magnetic measurements were performed by means of the vibrating sample magnetometery method, using a VSM 7407 magnetometer, at room temperature. Thermogravimetric analysis (TGA) was performed using a Perkin Elmer thermogravimetric analyzer. UV–Visible spectra in the 200–1000 nm range were obtained in DMF solvent on a Perkin Elmer Lambda 45 spectrophotometer. A Jenway model 4510 pH-meter was used for pH measurements by use of a combined electrode. An ultrasonication probe (Karl Deutsch, Germany) was used to disperse the nanoparticles in the solution.

### Preparation of magnetite nanoparticles (Fe_3_O_4_)

The Fe_3_O_4_ nanoparticles were prepared according to Ref.^45^ with minor modifications. Briefly, FeCl_3_⋅6H_2_O (11.68 g) and FeCl_2_⋅4H_2_O (4.30 g) were dissolved in 200 mL deionized water under nitrogen gas with vigorous stirring at 85 °C. Then, 20 mL of 30% aqueous ammonia was added to the solution. The color of the bulk solution changed from orange to black immediately. The magnetic precipitates were washed twice with deionized water and once with 0.02 mol/L sodium chloride. The washed magnetite was stored in deionized water at a concentration of 40 g/L.

### Preparation of Fe_3_O_4_@CS@Am nanocomposite

To a suspension of the Fe_3_O_4_ nanoparticles (0.35 g) in DI water/methanol (100 mL), chitosan (CS) (2 g) and acrylamide (1 g) were added. The mixed solution was ultrasonically dispersed for 30 min. The polymerization reaction of acrylamide was initiated by K_2_S_2_O_8_ (0.04 g), and the reaction was allowed to proceed for 12 h at 80 °C under nitrogen atmosphere and mechanical stirring. The resulting solid was magnetically separated, washed with water/methanol several times to remove the unreacted ligands and dried under vacuum.

### Preparation of Fe_3_O_4_@CS@Am@Nph nanocomposite

To a suspension of the Fe_3_O_4_@CS@Am nanoparticles (1 g) in ethanol (150 mL), 2-hydroxy-1-naphthaldehyde (Nph) was added (0.5 g). The reaction mixture was refluxed for 48 h under nitrogen atmosphere. The Fe_3_O_4_@CS@Am@Nph nanocomposite were separated by an external magnet, washed with distilled water and ethanol then dried in vacuum at 60 °C for 24 h.

### Adsorption experiments

Synthesized nanoparticles were used removal of everzol black dye from aqueous solutions. Various parameters such as initial concentration, contact time, adsorbent dose and pH on adsorption were studied. For performing the experiments, solution of 1000 mg/L of everzol black was prepared in deionized water and diluted to obtain the desired concentrations of dye. Different amounts of nanoparticles, varying from 20 to 100 mg, was suspended in a series of 40 mL dye solution with concentrations varying from 40 to 120 mg/L using 50 mL glass flasks. For suitable times from 10 to 20 min the suspensions were stirred and also the effect of solution pH on dye removal was investigated through adjusting by 0.01 N HCl or NaOH solutions. The nanoparticles adsorbent was separated from aqueous solution by an external magnetic field. The concentration of the everzol black was analyzed by UV-spectrophotometer at λ_max_ 600 nm. The amount of the dye adsorbed onto adsorbent (qe in mg/g) and the percentage of the dyes removed from the solution (R in %) were calculated from the equations:

10 $$\text{qe}=\frac{(\text{C}0-\text{Ce})}{\text{M}}\times \text{V},$$

11 $$\text{\%R}=\frac{(\text{C}0-\text{Ce})}{\text{C}0}\times 100,$$

where, C_0_ and C_e_ are the initial and equilibrium concentration of dye in solution (mg/L), respectively. V is the initial volume of the dye solution (L) and M is the mass of adsorbent used (g).

### Reusability studies

For the reusing possibility study, 20 mg of Fe_3_O_4_@CS@Am@Nph nanocomposite was added to the solution containing 25 mL of 100 mg/L dye for 30 min under 200 rpm at 298 K. The sample was filtered, and dye saturated Fe_3_O_4_@CS@Am@Nph sample was treated with of 0.1 M HCl. The percentage of desorption (D) was calculated using the equation:

12 $$\text{D}=\frac{{\text{Amount of dye desorbed}}}{{\text{Amount of dye adsorbed}}} \times 100.$$

## Supplementary Information

Supplementary Information.

## Data Availability

All data supporting the conclusions of this research article are included within the manuscript.
