# Supplementary material for: Preparation 2-hydroxy-1-naphthaldehyde cross-linked Fe3O4@chitosan-polyacrylamide nanocomposite for removal of everzol black from aqueous solutions
Source: Sci Rep. 2023 Jun 30;13:10618. doi: 10.1038/s41598-023-37243-5 (PMC10313670; doi:10.1038/s41598-023-37243-5)
Supplement: Supplementary file 1 — Supplementary Information. [file 41598_2023_37243_MOESM1_ESM.docx]

**Supporting Information for**

**Preparation 2-Hydroxy-1-naphthaldehyde cross-linked magnetic Fe_3_O_4_@chitosan-polyacrylamide nanocomposite for removal of everzol black from aqueous solutions**

Afshin Saadat^1, *^, Alireza Banaei^2^, Mehdi Sattarifar^2^, Parinaz Pargol Ghasemi^2^

^1^Department of Chemistry, Germi Branch, Islamic Azad University, Germi, Iran

^2^Department of Chemistry, Payame Noor University, Tehran, Iran, P.O. Box 19395-4697

***Corresponding Author Email:** [saadat@iaugermi.com](mailto:saadat@iaugermi.com)


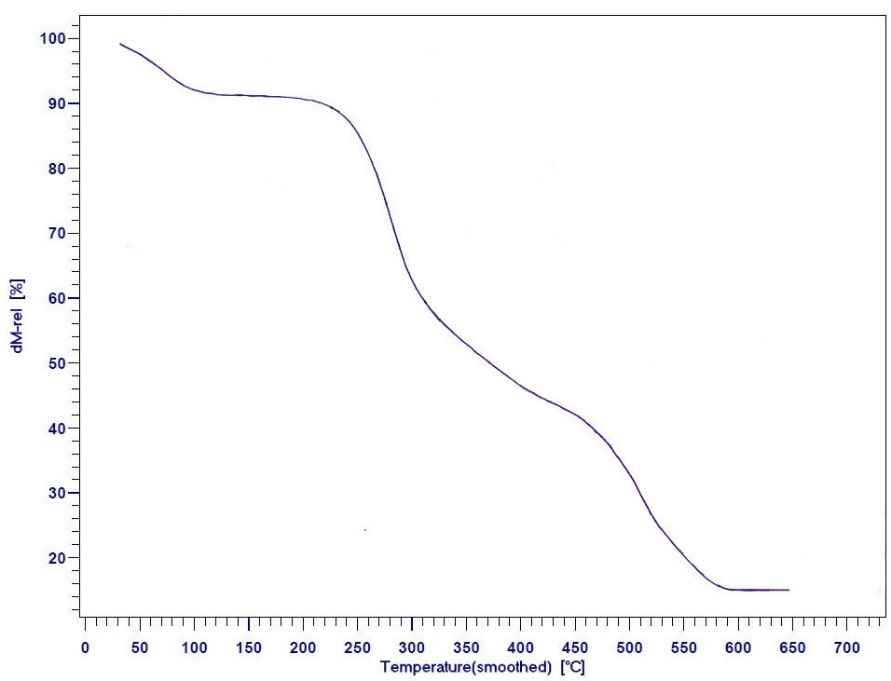


**Figure S1**. TGA curve of Fe_3_O_4_@CS@Am nanocomposite


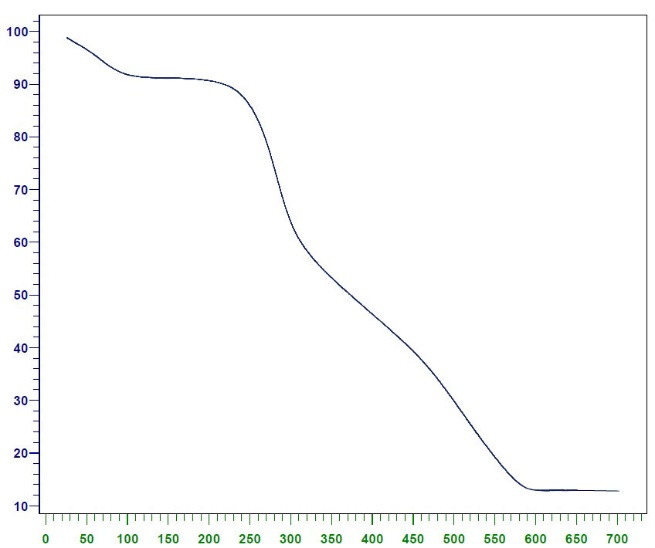


**Figure S2**. TGA curve of Fe_3_O_4_@CS@Am@Nph nanocomposite


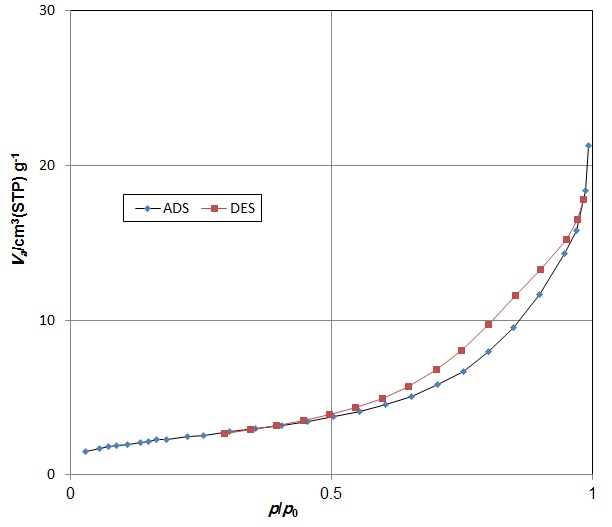


**Figure S3**. N_2_ adsorption-desorption isotherms of the Fe_3_O_4_@CS@Am@Nph nanocomposite

**Figure S4**. Chemical structure of Everzol Black

| Langmuir isotherm parameters | | | | Freundlich isotherm parameters | | | Temkin | | |
| --- | --- | --- | --- | --- | --- | --- | --- | --- | --- |
| q_m_ (mg/g) | K_L_ (L/mg) | R_L_ | R^2^ | K_F_ (L/mg) | n | R^2^ | A | B | R^2^ |
| 63.69 | 0.49 | 0.019 | 0.9959 | 28.87 | 3.38 | 0.9635 | 6.79 | 15.49 | 0.868 |

**Table S1.** Langmuir and Freundlich isotherms parameters and correlation coefficients for the adsorption of everzol black onto Fe_3_O_4_@CS@Am@Nph

| Dye | Temperature (K) | Parameters | | |
| --- | --- | --- | --- | --- |
|  |  | ∆G (kJ/mol) | ∆H (kJ/mol) | ∆S (kJ/mol) |
| everzol black | 283 | -0.97 | 2.42 | 0.012 |
|  | 293 | -1.09 |  |  |
|  | 308 | -1.27 |  |  |

**Table S2.** Thermodynamic parameters for the adsorption of everzol black on Fe_3_O_4_@CS@Am@Nph nanocomposite at different temperatures
